# Osteoporosis Beyond Awareness: Cross-National Differences in Preventive Deficits, Pharmacological Exposure, and Risk Clustering in Romania and Tunisia

**DOI:** 10.3390/diseases14070235

**Published:** 2026-06-30

**Authors:** Narcisa Jianu, Teodor Nicolae Onea, Dana Emilia Movilă, Valentina Oana Buda, Bianca Tot, Adina Nour, Silvia Luca, Diana Evelyne Buzzi, Laurențiu Brăescu, Minodora Andor

**Affiliations:** 1 Doctoral School, “Victor Babeş” University of Medicine and Pharmacy, 300041 Timişoara, Romania; narcisa.dinu@umft.ro (N.J.); bianca.tot@umft.ro (B.T.); adina.nour@umft.ro (A.N.); silvia.luca@umft.ro (S.L.); diana.mailat@umft.ro (D.E.B.); 2 Faculty of Mechanical Engineering, Department of Mathematics, Politehnica University of Timişoara, Victoriei Square 2, 300006 Timişoara, Romania; teodornicolae.onea@gmail.com; 3 Faculty of Medicine, “Victor Babeş” University of Medicine and Pharmacy, 2 Eftimie Murgu Street, 300041 Timisoara, Romania; braescu.laurentiu@umft.ro (L.B.); andor.minodora@umft.ro (M.A.); 4 Research Centre of Timisoara Institute of Cardiovascular Diseases, “Victor Babes” University of Medicine and Pharmacy, 300041 Timisoara, Romania; 5 Institute of Cardiovascular Diseases Timisoara, 13A Gheorghe Adam Street, 300310 Timişoara, Romania; 6 Faculty of Pharmacy, “Victor Babeş” University of Medicine and Pharmacy, 2 Eftimie Murgu Street, 300041 Timisoara, Romania; 7 Research Center for Pharmaco-Toxicological Evaluation, “Victor Babeș” University of Medicine and Pharmacy, Eftimie Murgu Sq. no. 2, 300041 Timișoara, Romania; 8 Multidisciplinary Heart Research Center, “Victor Babes” University of Medicine and Pharmacy, 2 Eftimie Murgu Sq., 300041 Timisoara, Romania

**Keywords:** bone disease, frailty, aging, knowledge, prevention measures, management, self-administered questionnaires, DXA screening, risk clustering, healthcare disparities

## Abstract

**Background:** Osteoporosis is increasingly understood as a complex population health condition shaped by interacting behavioral, metabolic, pharmacological, and healthcare system determinants rather than isolated skeletal risk factors. However, comparative studies integrating these dimensions across distinct healthcare and sociocultural settings remain scarce. We aimed to characterize cross-national differences in osteoporosis-related risk clustering between Romanian and Tunisian adults using an integrative multidimensional framework. **Methods:** We performed a comparative cross-sectional analysis of harmonized data from two pharmacy-based studies conducted in Romania and Tunisia, including adults aged ≥ 40 years (*n* = 349). Osteoporosis-related knowledge, lifestyle and metabolic risk factors, pharmacological exposures, preventive behaviors, and treatment patterns were assessed. Multivariable regression and mediation analyses were used to identify independent predictors of screening uptake and to evaluate the relationship between knowledge and preventive behavior. An exploratory cumulative preventive deficit score was used to estimate overall preventive burden within the pharmacy-based study sample. **Results:** Romanian participants demonstrated significantly higher osteoporosis knowledge than Tunisian participants (8.90 ± 2.20 vs. 7.83 ± 2.99; *p* < 0.001); however, knowledge was not independently associated with Dual-Energy X-ray Absorptiometry (DXA) uptake and did not mediate country-related differences in screening behavior. Compared with the Romanian cohort, the Tunisian cohort exhibited lower DXA screening rates (11.2% vs. 22.8%; *p* = 0.005), lower vitamin D supplementation (11.9% vs. 38.6%; *p* < 0.001), greater sedentary behavior, and a significantly higher cumulative preventive deficit burden (3.39 ± 1.08 vs. 2.77 ± 1.26; *p* < 0.001). Medication-related osteoporosis risk was also greater in Tunisia, particularly due to markedly higher corticosteroid exposure (7.5% vs. 0.5%; *p* = 0.002). Despite this less favorable preventive profile, the treatment gap among participants with diagnosed osteoporosis was significantly lower in Tunisia than in Romania (4.8% vs. 42.9%; *p* = 0.003). **Conclusions:** Distinct but convergent osteoporosis-related risk patterns were identified across the two populations, suggesting that osteoporosis vulnerability emerges through context-specific clustering of behavioral, pharmacological, and healthcare-access determinants rather than through isolated risk factors alone. The dissociation between knowledge and preventive behavior highlights the limited impact of awareness-based strategies when structural barriers remain unaddressed. These findings support a shift toward integrated, population-tailored osteoporosis prevention models that incorporate healthcare-system, medication-related, and behavioral determinants, in addition to conventional educational approaches.

## 1. Introduction

Osteoporosis is increasingly recognized not as an isolated skeletal disorder, but as part of a broader spectrum of age-related conditions characterized by multimorbidity and functional decline [1,2,3,4]. Beyond its direct impact on bone mineral density and fracture risk, osteoporosis frequently coexists with chronic diseases and metabolic disturbances, sharing common pathophysiological mechanisms such as chronic low-grade inflammation, oxidative stress, and hormonal dysregulation [5,6].

Emerging evidence supports the concept of a bone–vascular axis, highlighting the bidirectional relationship between bone metabolism and systemic health [7,8,9,10,11]. However, at the population level, this interaction is driven not only by biological mechanisms but also by the clustering of metabolic risk factors, lifestyle behaviors, and treatment-related exposures, which together shape heterogeneous risk profiles across populations [12,13].

In this context, factors such as body mass index, physical activity, and dietary habits represent key components of a broader cardiometabolic profile that may influence bone health [14,15,16]. Differences in these parameters across populations may reflect underlying socio-cultural patterns and contribute to variability in osteoporosis risk [2,17,18,19,20,21,22,23,24,25]. At the same time, long-term exposure to pharmacological treatments, including corticosteroids and other medications associated with bone loss, constitutes an important determinant of secondary osteoporosis, particularly in individuals with chronic comorbid conditions [26,27].

Importantly, these metabolic and pharmacological factors often coexist with behavioral determinants, such as a sedentary lifestyle, inadequate supplementation, and low uptake of screening strategies. The clustering of these elements may accelerate the progression toward frailty, a multidimensional syndrome characterized by increased vulnerability to adverse outcomes [28,29,30,31,32,33]. In this framework, osteoporosis can be interpreted not only as a disease of bone metabolism, but also as a marker of cumulative health deficits at the population level [28,34,35,36,37,38].

Despite the availability of effective diagnostic tools and preventive interventions, osteoporosis remains underdiagnosed and undertreated worldwide [39,40,41]. Although insufficient knowledge has been identified as a contributing factor, growing evidence suggests that awareness alone does not consistently translate into preventive behavior, highlighting the influence of structural and system-level determinants [42,43,44].

Comparative analyses across different healthcare systems provide a valuable opportunity to explore how these interconnected factors, such as metabolic risk, pharmacological exposure, and behavioral patterns, interact to shape osteoporosis-related outcomes [27,45,46]. However, such data remain limited, particularly in studies that integrate these dimensions across distinct socio-cultural contexts.

**Figure 1:**
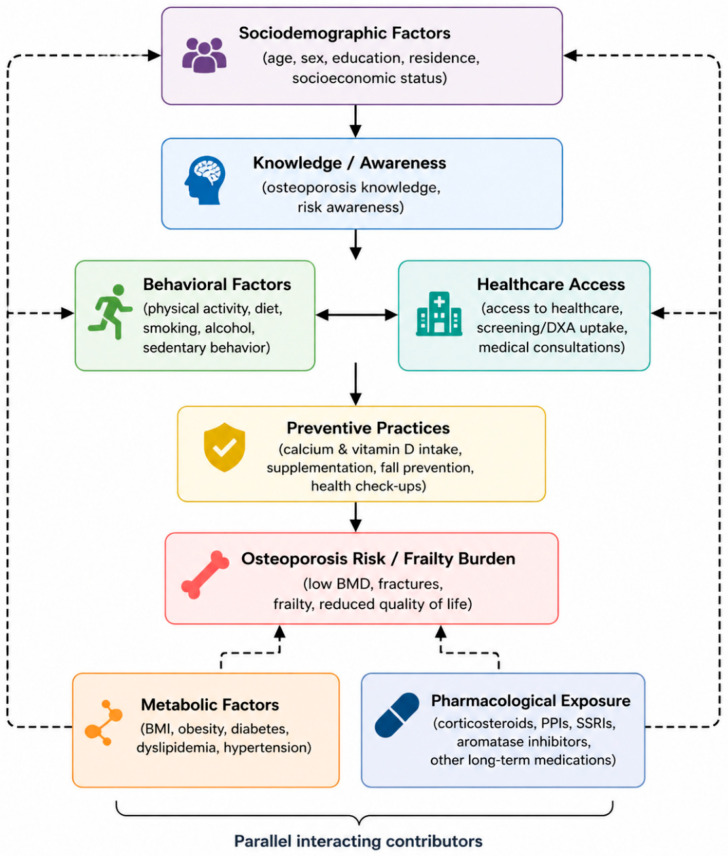
Integrative Population-Level Framework of Osteoporosis Risk Clustering. Abbreviations: BMI—body mass index; BMD—bone mineral density; DXA—dual-energy X-ray absorptiometry; PPIs—proton pump inhibitors; SSRIs—selective serotonin reuptake inhibitors.

Therefore, the aim of the present study was to apply an integrative, multidimensional framework to compare osteoporosis-related risk clustering between Romanian and Tunisian adults, incorporating knowledge, behavioral, metabolic, pharmacological, and preventive dimensions, and to examine their combined impact on potential implications for multimorbidity and frailty (Figure 1).

To our knowledge, this is the first cross-national comparative analysis that integrates knowledge of osteoporosis, behavioral risk factors, pharmacological exposure, and preventive deficits within a multimorbidity/frailty framework across Eastern European and North African populations.

While previous studies have typically examined osteoporosis awareness, lifestyle factors, or pharmacological risk exposures separately, few have adopted an integrative framework that conceptualizes osteoporosis risk as the result of clustered behavioral, metabolic, and treatment-related determinants operating within broader healthcare and sociocultural systems. Such an approach may better capture real-world osteoporosis vulnerability than analyses of isolated risk factors.

Therefore, the present study operationalizes a multidimensional framework of osteoporosis risk by translating theoretical determinants into measurable population-level indicators, including knowledge, preventive behavior, metabolic status, medication exposure, and healthcare access. Although the Romanian and Tunisian datasets have been previously reported separately, no study has directly compared these populations using a harmonized analytical framework. The present study extends previous findings by integrating both datasets into a unified comparative model, enabling the evaluation of cross-national differences in osteoporosis-related knowledge, preventive behaviors, medication-related risk, treatment gaps, and cumulative preventive deficits. In addition, the study introduces an exploratory cumulative preventive deficit score and applies multivariable regression and mediation analyses to investigate whether osteoporosis knowledge independently predicts screening behavior across different healthcare contexts.

## 2. Materials and Methods

### 2.1. Study Design and Conceptual Framework

The present analysis was structured around a multidimensional conceptual framework in which osteoporosis-related risk was operationalized as a clustered construct integrating five domains: sociodemographic determinants, metabolic profile, pharmacological exposure, lifestyle behaviors, and preventive healthcare engagement. It integrates data from two previously published investigations conducted in Romania and Tunisia [17,18]. The methodological framework was developed according to the study designs of Jianu et al. and Merlan et al., and further refined using internationally validated approaches for osteoporosis knowledge and prevention research [17,18]. More precisely, in the Romanian study, data were collected over a 3-month period (1 February to 30 April 2023) in 10 community pharmacies located in both urban and rural areas across four counties (Timiș, Arad, Caraș-Severin, and Olt). A structured, self-administered questionnaire was distributed to individuals visiting participating pharmacies, yielding 189 fully completed responses, which were included in the final analysis.

The Tunisian cohort was assessed using a comparable design, with data collected over a similar 3-month period in community pharmacies in Tunis. A validated self-administered questionnaire was distributed to pharmacy visitors, resulting in 160 completed questionnaires.

In both studies, pharmacies from different urban and rural settings were included, and data collection was performed by trained personnel following standardized procedures. The questionnaires were developed based on the international literature and were validated through expert review, pilot testing, and psychometric evaluation, as previously described in the original publications. For the purpose of the present analysis, the datasets were harmonized to ensure consistency in variable definitions and outcome measures across the two populations.

As mentioned, the study aimed to evaluate inter-population differences in osteoporosis-related knowledge, risk factors, and preventive behaviors, and to explore the underlying mechanisms influencing preventive decision-making.

### 2.2. Study Population and Data Sources

The pooled dataset included a total of 349 participants, derived from the Romanian cohort (*n* = 189) and the Tunisian cohort (*n* = 160). Participants were considered eligible for inclusion if they met the following criteria: age of 40 years or older, ability to understand and independently complete the study questionnaire, and provision of informed consent. Participants were excluded from the analysis if they had incomplete data for key variables or were unable to provide reliable responses due to language barriers or cognitive impairment.

### 2.3. Ethical Considerations

Both original cross-sectional studies included in the present comparative analysis were conducted under the supervision of the research team affiliated with the “Victor Babeș” University of Medicine and Pharmacy, Timișoara, Romania, and received approval from the Ethics Committee of the same institution (approval no. 47/2024). This approval covered data collection in both study settings. All participants provided written informed consent prior to participation.

### 2.4. Data Collection Instrument and Standardization

Data were collected using structured questionnaires developed and validated in the original studies, as previously described. The instruments were conceptually based on established osteoporosis assessment tools and included comparable domains across both cohorts.

The questionnaires captured information on socio-demographic characteristics, clinical profile, lifestyle behaviors, and preventive practices, alongside a multidimensional assessment of osteoporosis-related knowledge. A composite knowledge score was derived from items addressing disease definition, risk factors, prevention strategies, and management. The score was treated as a continuous variable. To ensure comparability, variables from both datasets were harmonized prior to analysis.

Moreover, data were collected by trained pharmacy personnel following standardized procedures. Participants were informed of the study objectives and provided written informed consent prior to completing the questionnaire. Data entry was performed using a double-entry procedure with independent verification to ensure accuracy.

To enable cross-national comparability, the Romanian and Tunisian datasets underwent a harmonization procedure prior to pooled analysis. The process was performed jointly by the investigators responsible for both original studies and was reviewed before statistical analysis. Because the two questionnaires were highly comparable in structure, content, and response format, most variables required no substantive recoding beyond standardization of variable names and coding conventions. Key domains included sociodemographic characteristics, osteoporosis-related knowledge, preventive practices, lifestyle behaviors, pharmacological exposure, osteoporosis diagnosis, and treatment status. Continuous variables (e.g., age, body mass index, and osteoporosis knowledge score) were calculated using identical methods in both datasets and therefore required no harmonization. Similarly, most categorical variables, including DXA screening, calcium and vitamin D supplementation, smoking status, physical activity, osteoporosis diagnosis, treatment status, and medication exposure, shared equivalent response structures and were retained without modification. The only notable harmonization step involved educational attainment, where slight differences in category structures between the two studies required recoding into unified analytic categories to ensure conceptual equivalence. A detailed overview of the harmonization process, including original variable wording, response categories, recoding procedures, final analytic coding, and missing data, is provided in Supplementary Table S1.

In addition, reliability and validation procedures were reviewed from the original studies. The Romanian questionnaire underwent expert review, pilot testing, and empirical validation. Internal consistency was assessed using Cronbach’s alpha (α = 0.60), while test–retest reliability was evaluated using the intraclass correlation coefficient (ICC = 0.65), indicating moderate reliability. Further details regarding questionnaire development and validation procedures are available in the original publications and should be considered when interpreting questionnaire-derived measures included in the pooled analysis.

### 2.5. Statistical Analysis

Statistical data processing was carried out using IBM SPSS Statistics v.26.0. For the analysis of mediation mechanisms, the PROCESS v4.2 macro developed by Andrew F. Hayes was used. The statistical significance threshold was set at *p* < 0.05 for all tests, corresponding to a 95% confidence interval (CI).

Categorical variables (socio-demographic data, risk factors, presence of diagnosis and treatment) were reported as absolute frequencies (*n*) and relative frequencies (percentages, %). The comparison of these variables between Romanian and Tunisian samples was performed using Pearson’s Chi-square test. In situations where the theoretical frequencies were reduced (below 5 units per cell), the Exact Fisher Test was applied to ensure the robustness of the results, especially in the analysis of the treatment gap. Continuous variables, such as age or knowledge scores, were expressed as mean and standard deviation (M ± SD).

To identify independent predictors of osteoporosis knowledge, a multiple linear regression model was fitted according to the following specification:

Knowledge Score = β_0_ + β_1_(Country) + β_2_(Age) + β_3_(Sex) + β_4_(Residence) + β_5_(Education) + β_6_(Family History of Osteoporosis) + ε,

where Knowledge Score represents the total osteoporosis knowledge score. Country was coded as Romania = 0 and Tunisia = 1; Sex as male = 0 and female = 1; Residence as rural = 0 and urban = 1; Family history of osteoporosis as no = 0 and yes = 1; Education was entered as an ordinal variable; and Age was treated as a continuous variable. The model was adjusted for all included covariates. Model performance was evaluated using the F statistic and adjusted R^2^. Unstandardized regression coefficients (B), standardized coefficients (β), and corresponding *p*-values were reported.

To identify independent predictors of DXA screening uptake, a binary logistic regression model was fitted according to the following specification:

logit(P(DXA)) = β_0_ + β_1_(Country) + β_2_(Age) + β_3_(Sex) + β_4_(Education) + β_5_(Family History of Osteoporosis) + β_6_(Knowledge Score),

where P(DXA) represents the probability of having undergone DXA screening. Country was coded as Romania = 0 and Tunisia = 1; Sex as male = 0 and female = 1; Family history of osteoporosis as no = 0 and yes = 1; Education was entered as an ordinal variable; and Knowledge Score was treated as a continuous variable. Overall model significance was assessed using the likelihood-ratio chi-square test. Model fit was evaluated using the Hosmer–Lemeshow goodness-of-fit test, while explanatory performance was assessed using the Nagelkerke pseudo-R^2^ statistic. Odds ratios (ORs) and 95% confidence intervals (CIs) were reported for all predictors. The mechanism by which the country of origin influences preventive behavior was explored using a mediation analysis (Model 4 in PROCESS), with the knowledge score as the mediating variable. The significance of the indirect effect was determined by the bootstrapping method with 5000 resamples, being considered valid only if the confidence interval (95% BootCI) did not include the value zero. The interaction between country and level of education was also tested to assess the equity of access to screening.

In the final stage, a Cumulative Preventive Deficit Score (CPDS) was constructed to provide an exploratory, integrative estimate of the modifiable preventive burden associated with osteoporosis prevention. The score was calculated by summing five preventive deficits: lack of DXA screening, absence of calcium supplementation, absence of vitamin D supplementation, sedentary lifestyle (<30 min/day of physical activity), and active smoking. These components were selected based on their established relevance to osteoporosis prevention and their frequent identification as modifiable behavioral or preventive determinants in the osteoporosis literature. Equal weighting was assigned to each component to preserve interpretability and avoid arbitrary assumptions regarding the relative contribution of individual deficits in the absence of validated weighting coefficients. Each component contributed 1 point, resulting in a total score ranging from 0 (no preventive deficits) to 5 (maximum preventive deficit burden). The CPDS should be interpreted as an exploratory composite preventive burden indicator rather than a validated clinical risk tool, as it has not been validated against clinical outcomes such as bone mineral density, fracture incidence, FRAX probability, falls, frailty measures, or other objective endpoints. Comparisons between countries were performed using the independent-samples *t*-test, with Welch correction applied when heteroscedasticity was detected by Levene’s test.

## 3. Results

The analysis of socio-demographic characteristics (Table 1) revealed significant structural differences between the two samples, especially in gender distribution, age categories, and educational level (*p* < 0.001).

In the Romanian sample, a marked female predominance was observed, with women accounting for 78.8% of participants, whereas in the Tunisian sample, the distribution was much more balanced, with men accounting for 44.4%. This finding reflects a marked difference in sex distribution between the two cohorts.

Regarding the age structure, although both groups showed similar shares in the under-60 age segment, there were notable differences at the extremes of the age pyramid. The group in Romania showed a more consistent presence of the 60–69 age segment (31.7%), but did not include participants aged 80 or older. In contrast, the Tunisian sample included a significant proportion of octogenarians (13.1%), indicating a greater representation of participants aged ≥ 80 years in the Tunisian cohort. Area of residence was the only demographic variable showing no statistically significant differences between countries (*p* = 0.141), with the majority of subjects coming from urban areas in both countries (69.3% in Romania and 61.2% in Tunisia).

The level of education configured contrasting profiles between the two populations, demonstrating different educational distributions between cohorts. While the Romanian cohort had a predominantly medium educational profile, with 51.9% of subjects graduating high school or post-secondary education, the Tunisian cohort showed a more pronounced polarization. Thus, the Tunisian sample had a higher share of people with university education (54.1%) than the Romanian one (34.9%), but also a double proportion of participants who completed only primary school (26.8% compared to 13.2% in Romania).

The analysis of risk factors and clinical history (Table 2) revealed significant differences between the two groups in terms of body mass index (BMI) distribution and family history of osteoporosis. In the Romanian sample, a much higher prevalence of overweight was observed, with 71.4% of the participants being classified as overweight or obese, compared to 48.2% in the Tunisian sample (*p* < 0.001). In contrast, the Tunisian group had a significantly higher share of people with normal weight (46.9% compared to 27% in Romania) and a more pronounced underweight category (5.0%).

Another important distinction was identified in the family history of osteoporosis (*p* = 0.002), a variable indicating a recognized genetic predisposition. Participants from Tunisia reported a significantly higher proportion of family members with the disease (31.2%) than the Romanian group (16.9%).

Regarding the acute clinical events and the established diagnosis, the samples showed relatively homogeneous profiles, with no major statistical differences. The prevalence of prior fractures (25.9% in Romania vs. 18.7% in Tunisia) and recent falls (24.3% in Romania vs. 18.7% in Tunisia) was comparable between the two countries (*p* > 0.05). No statistically significant differences were observed for fracture history or recent falls (*p* > 0.05). Additionally, the proportion of subjects who already had a formal diagnosis of osteoporosis was almost identical (14.8% in Romania and 13.1% in Tunisia, *p* = 0.757), as was the presence of associated pathologies, which affected about a third of the total participants in both groups.

Analysis of lifestyle behaviors (Table 3) revealed significant cultural and consumption differences between the two groups, particularly in relation to the use of stimulants and alcohol. While the prevalence of smoking was similar in both samples (approximately 22%, *p* = 0.797), alcohol and caffeine consumption showed major variations (*p* < 0.001). Participants from Romania reported much higher alcohol consumption (27.0% active drinkers) than those from Tunisia (3.7%). Furthermore, caffeine consumption was significantly higher in the Romanian sample (76.7%) than in the Tunisian sample (52.8%).

The level of daily physical activity showed a more sedentary profile in Tunisia, where 58.8% of participants self-reported less than 30 min of physical activity per day, compared to 45.5% in Romania (*p* = 0.005). Moreover, the proportion of people who practice sustained physical activity (over 60 min a day) is more than twice as high in the Romanian sample (23.8%) as in Tunisia (11.2%).

From the perspective of specific preventive measures, major deficits were observed in both groups, but with a more pronounced tendency towards lower uptake of preventive measures in the Tunisian cohort. Although calcium supplementation did not show a significant difference (approximately 24% globally; *p* = 0.081), vitamin D supplementation did (*p* < 0.001). In Romania, 38.6% of subjects use vitamin D supplementation, whereas in Tunisia, only 11.9% do. The same trend was confirmed in the case of screening by DXA investigation (*p* = 0.005), the testing rate being twice as high in the Romanian cohort (22.8%) compared to the Tunisian cohort (11.2%).

To assess differences in knowledge of osteoporosis between the two countries, a *t*-test for independent samples was applied (Table 4). The analysis revealed a statistically significant difference, with participants from Romania obtaining, on average, a higher knowledge score (M = 8.90, SD = 2.20) compared to participants from Tunisia (M = 7.83, SD = 2.99), *p* < 0.001. The effect size, assessed by Cohen’s d indicator, was 0.41. This result indicates a small to moderate difference in size between the two populations, suggesting that although the difference is statistically significant, the practical discrepancy in general knowledge is medium.

To control for possible confounders and assess independent predictors of osteoporosis knowledge, a multiple linear regression analysis was performed (Table 5). The independent variables in the model were: country of origin, age, gender, area of residence, level of education, and family history of osteoporosis.

The overall model was statistically significant (F(6, 339) = 6.010, *p* < 0.001), explaining 8% of the variance in the knowledge score (adjusted R^2^ = 0.080). In the multivariate analysis, the country of origin remained an extremely significant independent predictor (B = −1.165, *p* < 0.001), indicating that the association between cohort and knowledge score remained statistically significant after adjustment for the variables included in the model. Additionally, education level (B = 0.704, *p* = 0.001) and family history of osteoporosis (B = 1.034, *p* = 0.002) were independent determinants of higher knowledge levels. In contrast, gender, age, and area of residence were not significant independent predictors in this integrated model (*p* > 0.05).

The analysis of standardized coefficients (Beta) indicates that country of origin (Beta = −0.219) and level of education (Beta = 0.201) had the strongest influence on the knowledge score, followed by family history (Beta = 0.166).

To identify factors influencing the probability of undergoing the DXA screening, a binary logistic regression model was run (Table 6). The model included knowledge score, country, age, gender, education level, and family history as independent variables.

The overall model was statistically significant (χ^2^(7) = 39.330, *p* < 0.001) and showed an adequate fit to the data, according to the Hosmer–Lemeshow test (*p* = 0.260). The included variables explained 17.7% of the variance in the decision to perform the DXA test (R^2^ Nagelkerke = 0.177).

The analysis revealed that age, gender, family history, and country of origin are significant independent predictors for screening. Thus, for each additional year of age, the chance of performing a DXA test increases by 6.5% (OR = 1.065, 95% CI: 1.032–1.099). Women were 3.4 times more likely to get tested than men (OR = 3.425, *p* = 0.003), and having a family history of osteoporosis increased the odds by almost 3 times (OR = 2.906, *p* = 0.002).

Regarding regional differences, patients in Tunisia were significantly less likely to undergo DXA testing than those in Romania (OR = 0.407, *p* = 0.015), indicating that cohort membership remained independently associated with self-reported DXA screening uptake after adjustment for the variables included in the model. A particularly important finding is that the level of education and knowledge score about osteoporosis were not significant predictors (*p* > 0.05) of the investigation’s performance, suggesting that higher knowledge scores were not independently associated with self-reported DXA screening uptake in the adjusted model.

To test the hypothesis of possible inequalities in access to screening, an interaction term between country of origin and level of education was included in the model. The analysis indicated that the interaction was not statistically significant (*p* = 0.301), suggesting that the association between education level and DXA test performance does not differ significantly between Romania and Tunisia.

To investigate the mechanisms underlying preventive behavior, the hypothesis that knowledge of osteoporosis (mediator) explains the relationship between country of origin (independent variable) and the performance of the DXA test (dependent variable) was tested. The mediation analysis was performed using the PROCESS macro (Model 4), based on 5000 bootstrap samples, controlling for confounding factors (age, gender, education, and family history).

**Figure 2:**
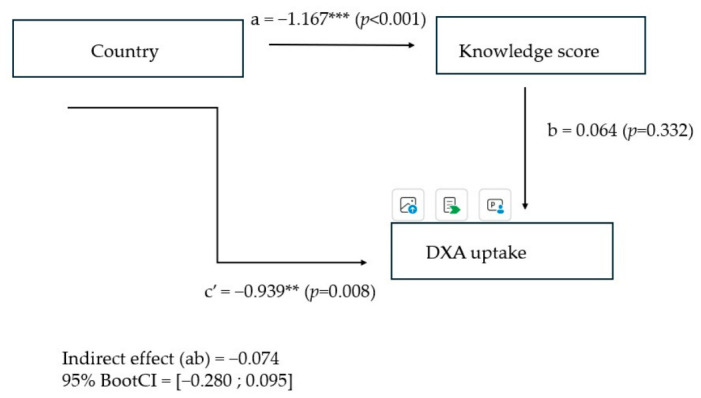
Exploratory mediation model assessing the role of osteoporosis knowledge in the association between country of origin and DXA screening uptake. Values represent unstandardized regression coefficients. The indirect effect was estimated using 5000 bootstrap samples (95% BootCI). ** indicates *p* < 0.01; *** indicates *p* < 0.001.

The results showed that country of origin significantly predicts knowledge level (B = −1.167, *p* < 0.001), but knowledge does not influence the probability of performing DXA screening (B = 0.064, *p* = 0.332). The country’s direct effect on the screening decision remained statistically significant (Direct effect = −0.939, *p* = 0.008). Indirect effect testing yielded −0.074, with a bootstrap confidence interval of −0.280 to 0.095. Since this confidence interval includes zero, the indirect effect is not statistically significant. Therefore, the exploratory mediation analysis (Figure 2) did not support a statistically significant indirect association between cohort and self-reported DXA screening uptake through osteoporosis knowledge.

To assess differences in the treatment gap between the two populations (Table 7), the analysis was restricted to patients who reported a confirmed diagnosis of osteoporosis (*n* = 49). Due to the small size of this sub-sample and the low frequency of events of interest (diagnosed but untreated patients), the multivariate approach was avoided to prevent instability in the statistical model. Instead, proportional differences were analyzed using the Fisher Exact Test. As shown in Table 7, among participants reporting a confirmed osteoporosis diagnosis, untreated disease was more frequently reported in the Romanian cohort than in the Tunisian cohort: in Tunisia, only 4.8% of diagnosed patients (*n* = 1) were not following a specific treatment, compared to 42.9% in Romania (*n* = 12). The Exact Fisher test showed that this difference in non-initiation or discontinuation of treatment is statistically significant (*p* = 0.003).

**Figure 3:**
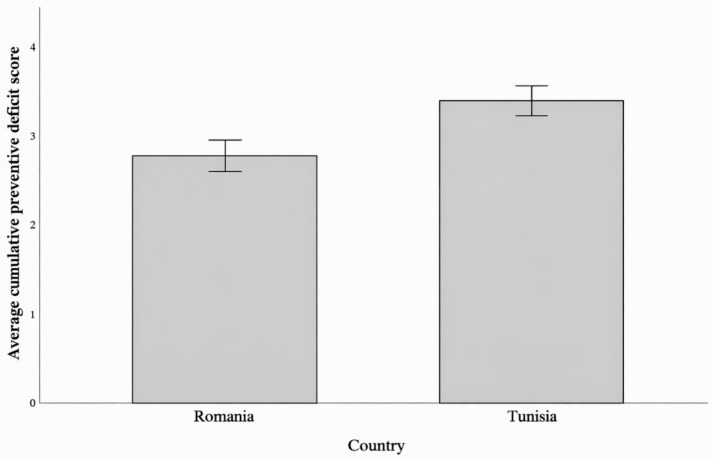
Comparison of the Cumulative Preventive Deficit Score (CPS) between the samples from Romania and Tunisia.

Finally, the overall preventive profile was compared using an exploratory cumulative preventive deficit score (Table 8), calculated as the sum of five modifiable preventive deficits (absence of DXA screening, absence of calcium supplementation, absence of vitamin D supplementation, physical inactivity, and smoking). The score ranged from 0 (no preventive deficits) to 5 (maximum preventive deficit burden). The independent-samples *t*-test revealed a statistically significant difference between cohorts (t(346.98) = −4.916, *p* < 0.001). Participants from the Tunisian cohort exhibited higher cumulative preventive deficit scores (M = 3.39, SD = 1.08) than participants from the Romanian cohort (M = 2.77, SD = 1.26), indicating a greater accumulation of preventive deficits within this sample (Figure 3).

As shown in Table 9, a significantly higher proportion of Tunisian participants (38.8%) were undergoing chronic treatment in these classes than Romanian participants (23.8%), a statistically significant difference (χ^2^ = 8.41; df = 1; *p* = 0.004). At the level of the pharmacological profile analyzed by sub-classes, the only difference that reaches statistical significance is corticosteroid therapy, which is more than ten times more prevalent in the Tunisian sample (7.5% vs. 0.5%, *p* = 0.002). For the other classes (antiepileptics, antitumors, antidepressants), the proportions are numerically higher in Tunisia but do not reach the *p* < 0.05 threshold, and PPI treatment has an almost identical prevalence in both countries (≈18.5%). The overall test across all six categories is significant (χ^2^ = 21.13; df = 5; *p* < 0.001), but this result is substantially influenced by differences in corticosteroid therapy and should be interpreted with caution due to the expected low frequencies in some cells.

The Tunisian cohort demonstrated significantly higher exposure to medications potentially associated with osteoporosis risk, primarily driven by corticosteroid use (7.5% vs. 0.5%, *p* = 0.002)—one of the most important causes of secondary osteoporosis. For the other classes, differences between countries did not reach statistical significance at the individual level, but the overall profile remains distinct between the two populations.

Table 10, Table 11 and Table 12 summarize the relationships between information sources and osteoporosis knowledge. Patients who indicated the doctor as their main source of information had a higher average knowledge score than those informed by other sources, both globally and in each country. However, the difference did not reach statistical significance (*p* > 0.05) in any of the three analyses.

The analysis shows statistically significant differences globally (*p* = 0.003) and in the Romanian sample (*p* = 0.020) between patients who received information from the doctor and those who did not. In Tunisia, the difference remains insignificant (*p* = 0.093). The proportion of patients who received information from the physician did not differ significantly between countries (39.2% RO vs. 33.1% TN; χ^2^ = 1.11; *p* = 0.292).

Of the 49 patients with confirmed osteoporosis in the 50-year-old subgroup (28 RO, 21 TN), the treatment gap remained statistically significant: 42.9% of diagnosed Romanians were not receiving specific treatment, compared to only 4.8% in Tunisia (Fisher’s exact test, *p* = 0.003). As summarized in Table 13, the combined analysis of risk factors, preventive behaviors, medication-related exposures, and treatment status indicates distinct osteoporosis-related risk clustering patterns across the two cohorts.

## 4. Discussion

The principal contribution of this study lies not merely in comparing two previously published studies, but in integrating their datasets into a harmonized analytical framework that enables direct cross-national comparisons and the investigation of research questions that could not be evaluated in either study independently. Its strongest contribution is the demonstration that higher osteoporosis knowledge did not translate into DXA screening behavior, while preventive deficits and medication-related risk profiles differed substantially between the two pharmacy-based cohorts. Moreover, the study provides four novel elements: (i) a unified comparison of osteoporosis-related preventive profiles between Romania and Tunisia, (ii) multivariable evaluation of factors associated with DXA uptake, (iii) an exploratory mediation analysis indicating that osteoporosis knowledge did not significantly mediate the association between country and DXA screening uptake, and (iv) the development of an exploratory cumulative preventive deficit score to characterize the accumulation of modifiable preventive deficits at the population level. Our findings suggest that distinct population-specific constellations of metabolic, pharmacological, and behavioral exposures may converge on similar osteoporosis-related adverse outcomes through different combinations of these factors. These findings build upon our previously published Romanian study [17], which emphasized underdiagnosis, treatment gaps, and frailty-related implications, and complement the Tunisian study [18], which reported suboptimal awareness, low screening uptake, and insufficient preventive practices at the population level. For clarity, the present findings can be interpreted through five interconnected domains: osteoporosis knowledge, screening behavior, metabolic and pharmacological risk profiles, treatment gap, and cumulative preventive deficits.

A first relevant finding is the significantly higher osteoporosis-related knowledge observed in the Romanian cohort, with education level and family history emerging as independent predictors. This pattern is consistent with the Tunisian study, which found that higher knowledge scores were associated with higher education, urban residence, and prior exposure to the disease. Similar associations have been widely reported in the literature, including studies from Poland and Saudi Arabia, where educational attainment consistently predicts better osteoporosis awareness [47,48,49,50,51,52]. These findings reinforce the importance of socio-demographic determinants in shaping health literacy across populations.

However, the most important observation of the present study is that higher knowledge did not translate into improved preventive behavior. Knowledge score was not independently associated with DXA screening, and mediation analysis confirmed that knowledge did not explain the relationship between country and screening uptake. This finding is strongly supported by previous research. Studies conducted in Malaysia, Saudi Arabia, and India have consistently reported moderate or even good levels of knowledge, but poor adherence to preventive behaviors, including low levels of physical activity, inadequate supplementation, and limited screening uptake [19,22,50,52,53,54,55,56,57,58]. Collectively, these results suggest that awareness alone is insufficient to drive behavioral change and that structural, economic, and healthcare-related barriers play a major role in shaping preventive practices. Given the cross-sectional nature of the study, the mediation analysis should be interpreted as exploratory and hypothesis-generating rather than causal, since temporal relationships between knowledge, physician contact, diagnosis, and screening behavior cannot be established. Consequently, the directionality of these associations remains uncertain, and osteoporosis-related knowledge may have resulted from prior healthcare interactions rather than acting as a precursor of screening behavior.

This interpretation is further supported by the marked differences observed in screening behavior. DXA uptake was significantly lower in Tunisia, and the country of origin remained an independent predictor even after adjustment for demographic variables. These findings are directly aligned with the Tunisian study [18], where only a small proportion of participants had undergone DXA testing despite a non-negligible burden of fractures and osteoporosis. The authors highlighted limited access to diagnostic infrastructure and delayed diagnosis as key contributing factors. Consistent with these observations, the disparities observed between cohorts may reflect differences in healthcare access, healthcare system organization, and other unmeasured contextual factors. However, given the observational design and baseline differences between samples, these interpretations should be considered hypothesis-generating rather than definitive explanations of the observed associations. The metabolic dimension of risk also differed substantially between the two populations. The Romanian cohort showed a higher prevalence of overweight and obesity, whereas the Tunisian cohort was characterized by a more sedentary lifestyle and a higher cumulative preventive deficit. These findings suggest distinct cardiometabolic profiles that may influence bone health through different pathways. While excess body weight has traditionally been considered protective against osteoporosis, recent evidence indicates that obesity may negatively affect bone quality and increase fracture risk through metabolic dysregulation [45,59]. On the other hand, physical inactivity, more prevalent in the Tunisian cohort, is a well-established contributor to bone loss and reduced muscle strength, thereby increasing the risk of falls and fractures [60,61]. Similar patterns have been reported in Lebanon and Saudi Arabia, where sedentary behavior and poor lifestyle habits were strongly associated with inadequate osteoporosis prevention [21,53,56,62,63]. Nevertheless, these findings should be interpreted in light of the demographic differences between cohorts, particularly regarding age and sex distribution.

A particularly relevant finding of the present study is the substantially higher burden of medication-related osteoporosis risk observed in the Tunisian cohort, as self-reported by the respondents. Beyond the markedly increased prevalence of chronic corticosteroid exposure, Tunisian participants also demonstrated a numerically higher prevalence of several other medication classes associated with impaired bone health, including antiepileptics, antidepressants, and anticancer therapies. Collectively, these findings suggest a broader pharmacological risk profile consistent with increased vulnerability to secondary osteoporosis. This observation is clinically important because medication-related bone loss remains one of the most under-recognized contributors to osteoporosis, particularly in patients with chronic inflammatory, neurological, oncological, or psychiatric conditions [59,64,65,66]. In addition to direct skeletal effects, polypharmacy may further contribute indirectly to fracture risk through impaired balance, sedation, falls, and reduced treatment adherence. Thus, pharmacological exposure may represent both a marker of chronic disease burden and an independent contributor to osteoporosis-related vulnerability [67]. When interpreted alongside the higher prevalence of chronic treatment overall, these findings support the hypothesis that the Tunisian cohort may present a more pronounced multimorbidity-associated osteoporosis risk profile, in which chronic disease burden and treatment-related skeletal effects interact with behavioral and preventive deficits [59,64]. Previous studies have consistently identified long-term corticosteroid use as a major contributor to bone loss, while recent evidence suggests that overall medication burden (polypharmacy) may also be associated with reduced bone mineral density [64,67]. In this context, the higher pharmacological exposure observed in the Tunisian cohort may reflect a greater burden of chronic disease and multimorbidity, contributing to increased osteoporosis risk beyond traditional lifestyle factors. However, information on dose, duration, route of administration, and treatment indication was not available. Consequently, these findings should be interpreted as indicators of potential medication-related osteoporosis risk rather than direct estimates of pharmacologically induced bone loss.

The role of comorbidities further supports this interpretation. Although no significant difference in the overall prevalence of associated pathologies was observed between the two groups in our analysis, the Tunisian study [18] demonstrated a strong association between comorbid conditions and osteoporosis diagnosis. Similarly, studies conducted in clinical settings have reported frequent coexistence of osteoporosis with metabolic and chronic diseases, supporting the concept of osteoporosis as part of a broader multimorbid phenotype [59,68]. This perspective is particularly relevant in aging populations, where multiple risk factors converge and interact.

Another noteworthy finding concerns the role of healthcare professionals as a source of information. In our study, receiving information from a physician was associated with significantly higher knowledge scores, particularly in the Romanian cohort [17]. This observation is consistent with the Tunisian study [18], where physicians were identified as a primary source of osteoporosis-related information, and with other studies showing that direct interaction with healthcare providers improves patient awareness [63,68]. These findings highlight the importance of integrating educational interventions into routine clinical practice.

The treatment gap identified in our analysis adds an additional layer of complexity. Among participants with confirmed osteoporosis, untreated disease was significantly more frequent in the Romanian cohort than in the Tunisian cohort. This finding appears paradoxical when considering the poorer preventive profile observed in Tunisia; however, it is consistent with the Tunisian study [17], where a high proportion of diagnosed patients received treatment, predominantly bisphosphonates. These findings may suggest that preventive deficits and treatment gaps occur at different stages of the osteoporosis care pathway across the two cohorts [17,18]. Beyond individual-level factors, healthcare-system characteristics may also contribute to the observed differences in treatment gaps. Variations in reimbursement policies, medication affordability, referral pathways, and the involvement of primary care physicians in osteoporosis management may influence whether patients initiate and maintain treatment after diagnosis. Although these factors were not directly assessed in the present study, they may partly explain why treatment deficits appeared to occur at different stages of the osteoporosis care pathway. Future research incorporating healthcare system indicators would be valuable for clarifying the contribution of structural and organizational determinants to osteoporosis management. This distinction is consistent with previous reports highlighting the persistence of treatment gaps in osteoporosis management at different stages of care [39,59] and underscores the importance of designing targeted interventions adapted to different healthcare settings. Taken together, these findings support a model of osteoporosis risk characterized by the clustering of metabolic, pharmacological, and behavioral factors. Within the studied pharmacy-based samples, the Romanian cohort was characterized by a higher prevalence of overweight and obesity, together with a larger post-diagnostic treatment gap, whereas the Tunisian cohort demonstrated lower screening uptake, lower supplementation rates, higher sedentary behavior, and greater pharmacological exposure. Although these patterns differed between cohorts, they may represent distinct constellations of risk factors associated with osteoporosis-related vulnerability. Despite these differences, both profiles may contribute to similar adverse outcomes, including underdiagnosis, insufficient prevention, and a greater overall burden of osteoporosis-related risk [45,60].

From a clinical and public health perspective, these results suggest that strategies to improve osteoporosis outcomes should go beyond educational interventions and address broader determinants of health. Improving access to screening, optimizing chronic disease management, reviewing long-term medication use, and promoting sustainable lifestyle changes are essential components of an integrated approach [39,59]. Importantly, interventions should be tailored to population-specific risk profiles, accounting for differences in healthcare systems and socio-cultural contexts. Nevertheless, the present findings should be interpreted as associations observed within two pharmacy-based cohorts and should not be extrapolated as direct evidence of national-level differences.

This study has several strengths, including its comparative design and the integration of multiple dimensions of osteoporosis-related risk. However, several limitations should be acknowledged.

First, the cross-sectional design precludes causal inference and limits interpretation of temporal relationships between knowledge, preventive behaviors, and screening uptake. Second, data were based on self-reported questionnaires, which may introduce recall bias, reporting bias, and misclassification, particularly regarding fracture history, osteoporosis diagnosis, medication use, and preventive practices. Third, participants were recruited exclusively from community pharmacies. Consequently, the study population may overrepresent healthcare-engaged individuals, medication users, and persons with existing chronic conditions, potentially introducing selection bias and limiting the generalizability of the findings to the broader populations of Romania and Tunisia. This sampling approach may have led to higher levels of health awareness, healthcare utilization, and preventive behaviors than would be expected in the general population. Therefore, the present study should be interpreted as a pharmacy-based comparative cross-sectional analysis rather than a population-representative comparison.

In addition, the present analysis represents a secondary pooled evaluation of two previously independent studies. Although substantial efforts were made to harmonize variables across datasets, residual differences in questionnaire interpretation, cultural context, or implementation procedures cannot be fully excluded. Furthermore, important baseline differences existed between cohorts, particularly regarding sex distribution, age structure, and educational attainment. Although selected variables were adjusted for in multivariable analyses, residual confounding may still have influenced some of the observed between-country differences.

Finally, important structural differences between the Romanian and Tunisian healthcare systems, including reimbursement policies, diagnostic infrastructure, and access to specialist care, may have influenced screening and treatment behaviors independently of the individual-level determinants measured in this study.

Nevertheless, the study provides valuable real-world comparative insights into osteoporosis-related knowledge, preventive practices, medication-related risk, and treatment patterns across two distinct healthcare and sociocultural settings.

## 5. Conclusions

Overall, the findings support a multidimensional model of osteoporosis risk in which disease vulnerability reflects clustered and context-dependent combinations of behavioral, metabolic, pharmacological, and healthcare-access determinants.

Although higher levels of knowledge were observed in the Romanian cohort, this did not translate into improved preventive behaviors, confirming that awareness alone is insufficient to drive effective osteoporosis prevention. The persistence of low screening uptake and suboptimal supplementation, particularly in the Tunisian cohort, underscores the importance of structural barriers, including access to diagnostic services and healthcare organizations.

Importantly, our findings suggest that osteoporosis-related vulnerability may arise from clustered patterns of metabolic, behavioral, and pharmacological determinants that differ across cohorts. Within the studied pharmacy-based samples, the Romanian cohort demonstrated a higher prevalence of overweight and obesity, together with a larger post-diagnostic treatment gap, whereas the Tunisian cohort was characterized by lower screening uptake, lower supplementation rates, higher sedentary behavior, and greater medication-related osteoporosis risk, particularly corticosteroid exposure.

These distinct but converging risk profiles highlight the need for tailored, population-specific strategies that go beyond education alone. Effective interventions should integrate improved access to screening, optimization of chronic disease management, rational pharmacotherapy, and promotion of sustainable lifestyle changes. The observed differences between cohorts also suggest distinct priorities for public health interventions. In the Romanian cohort, efforts should focus on reducing post-diagnostic treatment gaps by improving treatment initiation, long-term adherence, and continuity of osteoporosis care after diagnosis. In contrast, the Tunisian cohort may benefit more from strategies aimed at increasing DXA screening uptake, promoting physical activity and vitamin D supplementation, and strengthening medication review practices in primary care, particularly regarding long-term corticosteroid exposure. These findings support the need for healthcare-system-specific approaches rather than uniform osteoporosis prevention strategies.

Taken together, the findings emphasize the importance of addressing osteoporosis prevention through integrated approaches that consider preventive behaviors, screening practices, treatment patterns, and medication-related risk factors rather than relying exclusively on educational interventions. Although frailty, fracture incidence, and clinical outcomes were not directly assessed, the observed clustering of behavioral, metabolic, and pharmacological determinants supports the concept that osteoporosis may be embedded within broader patterns of multimorbidity and age-related vulnerability. Future studies incorporating clinical, functional, and frailty-related outcomes are warranted to further investigate these relationships.

## Figures and Tables

**Table 1:** Socio-demographic characteristics of the samples.

	Romania (*n* = 189)	Tunisia (*n* = 160)	Total (*n* = 349)	*p*
Gender, *n* (%)				<0.001 *
Male	40 (21.2%)	71 (44.4%)	111 (31.8%)	
Female	149 (78.8%)	89 (55.6%)	238 (68.2%)	
Age category, *n* (%)				<0.001 *
<50	40 (21.2%)	36 (22.5%)	76 (21.8%)	
50–59	58 (30.7%)	48 (30.0%)	106 (30.4%)	
60–69	60 (31.7%)	31 (19.4%)	91 (26.1%)	
70–79	31 (16.4%)	24 (15.0%)	55 (15.8%)	
>80	0 (0.0%)	21 (13.1%)	21 (6.0%)	
Area of residence, *n* (%)				0.141
Rural	58 (30.7%)	62 (38.8%)	120 (34.4%)	
Urban	131 (69.3%)	98 (61.2%)	229 (64.6%)	
Level of education, *n* (%)				<0.001 *
Elementary School	25 (13.2%)	42 (26.8%)	67 (19.4%)	
High School/Post-secondary studies	98 (51.9%)	30 (19.1%)	128 (37.0%)	
Higher education	66 (34.9%)	85 (54.1%)	151 (43.6%)	

Legend: * *p* < 0.05.

**Table 2:** Risk factors, history, and clinical data.

	Romania (*n* = 189)	Tunisia (*n* = 160)	Total (*n* = 349)	*p*
BMI categories, *n* (%)				<0.001 *
Under weight	3 (1.6%)	8 (5.0%)	11 (3.2%)	
Normal weight	51 (27.0%)	75 (46.9%)	126 (36.1%)	
Over weight	84 (44.4%)	51 (31.9%)	135 (38.7%)	
Obesity	51 (27.0%)	26 (16.3%)	77 (22.1%)	
Family history of osteoporosis, *n* (%)				0.002 *
No	157 (83.1%)	110 (68.8%)	267 (76.5%)	
Yes	32 (16.9%)	50 (31.2%)	82 (23.5%)	
Recent Fall, *n* (%)				0.242
No	143 (75.7%)	130 (81.3%)	273 (78.2%)	
Yes	46 (24.3%)	30 (18.7%)	76 (21.8%)	
Fractures in history, *n* (%)				0.124
No	140 (74.1%)	130 (81.3%)	270 (77.4%)	
Yes	49 (25.9%)	30 (18.7%)	79 (22.6%)	
Diagnosis of osteoporosis, *n* (%)				0.757
No	161 (85.2%)	139 (86.9%)	300 (86.0%)	
Yes	28 (14.8%)	21 (13.1%)	49 (14.0%)	
Associated pathologies, *n* (%)				0.486
No	134 (70.9%)	107 (66.9%)	241 (69.1%)	
Yes	55 (29.1%)	53 (33.1%)	108 (30.9%)	

Legend: * *p* < 0.05.

**Table 3:** Lifestyle and prevention.

	Romania (*n* = 189)	Tunisia (*n* = 160)	Total (*n* = 349)	*p*
Smoking, *n* (%)				0.797
No	148 (78.3%)	123 (76.9%)	271 (77.7%)	
Yes	41 (21.7%)	37 (23.1%)	78 (22.3%)	
Alcohol consumption, *n* (%)				<0.001 *
No	129 (68.3%)	150 (93.8%)	279 (79.9%)	
Occasional	9 (4.7%)	4 (2.5%)	13 (3.7%)	
Yes	51 (27.0%)	6 (3.7%)	57 (16.4%)	
Caffeine consumption, *n* (%)				<0.001 *
No	44 (23.3%)	75 (47.2%)	119 (34.2%)	
Yes	145 (76.7%)	84 (52.8%)	229 (65.8%)	
Daily physical activity, *n* (%)				0.005 *
Less than 30 min/day	86 (45.5%)	94 (58.8%)	180 (51.57%)	
30–60 min/day	58 (30.7%)	48 (30.0%)	106 (30.4%)	
More than 60 min/day	45 (23.8%)	18 (11.2%)	63 (18.2%)	
Calcium supplements, *n* (%)				0.081
No	135 (71.4%)	128 (80.0%)	263 (75.4%)	
Yes	54 (28.6%)	32 (20.0%)	86 (24.6%)	
Vitamin D supplements, *n* (%)				<0.001 *
No	116 (61.4%)	141 (88.1%)	257 (73.6%)	
Yes	73 (38.6%)	19 (11.9%)	92 (26.4%)	
DXA, *n* (%)				0.005 *
No	146 (77.2%)	142 (88.8%)	288 (82.5%)	
Yes	43 (22.8%)	18 (11.2%)	61 (17.5%)	

Legend: * *p* < 0.05.

**Table 4:** Overall Knowledge Score.

	Romania (*n* = 189)	Tunisia (*n* = 160)	Total (*n* = 349)	*p*
Knowledge Score, mean ± DS				<0.001 *
	8.90 ± 2.20	7.83 ± 2.99	8.41 ± 2.65	

Legend: * *p* < 0.05.

**Table 5:** Multiple linear regression analysis for osteoporosis knowledge score predictors.

Predictive Variable	B	ES	Beta	t	*p*
(Constant)	6.351	1.151		5.519	<0.001 *
Country (Romania vs. Tunisia)	−1.165	0.289	−0.219	−4.028	<0.001 *
Age (years)	0.009	0.013	0.038	0.652	0.515
Gender (Male vs. Female)	0.377	0.314	0.066	1.201	0.230
Area of residence (Rural vs. Urban)	0.013	0.298	0.002	0.043	0.965
Level of education	0.704	0.210	0.201	3.358	0.001 *
Family history of osteoporosis	1.034	0.329	0.166	3.143	0.002 *
Model Summary: R^2^ = 0.096; R^2^ Adjusted = 0.080; F(6, 339) = 6.010, *p* < 0.001					

Legend: B = Non-standard coefficient; ES = standard error of the non-standard coefficient; Beta = standardized coefficient; t = the value of the test t; *p* = level of statistical significance; * *p* < 0.05.

**Table 6:** Binary logistic regression model predicting self-reported DXA screening uptake.

Predictive Variable	B	ES	Wald	*p*	OR	IC 95% for OR
Knowledge score	0.063	0.066	0.906	0.341	1.065	0.936–1.212
Country (Tunisia vs. Romania)	−0.898	0.370	5.910	0.015 *	0.407	0.197–0.840
Age (years)	0.063	0.016	15.121	<0.001 *	1.065	1.032–1.099
Gender (Female vs. Male)	1.231	0.417	8.714	0.003 *	3.425	1.512–7.755
Level of education (vs. Elementary School)			2.109	0.348		
High School/Post-Secondary Education	0.481	0.458	1.102	0.294	1.618	0.659–3.974
Higher education	0.706	0.486	2.109	0.146	2.026	0.781–5.254
Family history (Yes vs. No)	1.067	0.347	9.460	0.002 *	2.906	1.473–5.736
Constant	−7.314	1.462	25.037	<0.001 *	0.001	
Model performance: R^2^ Nagelkerke = 0.177; Hosmer–Lemeshow test: *χ*^2^(8) = 10.076, *p* = 0.260						

Legend: B = Non-standard coefficient; ES = standard error; Wald = The value of the test Wald; OR = Odds Ratio; IC = Confidence interval. The reference group for categorical variables is coded with 0 (Romania, Male, Elementary school, No family history); * *p* < 0.05.

**Table 7:** Comparison of treatment gap among participants with confirmed osteoporosis diagnosis.

	Receiving Osteoporosis Treatment			
	Yes (*n* = 36)	No (*n* = 13)	Total (*n* = 49)	*p*
Country, *n* (%)				0.003 *
Romania	16 (57.1%)	12 (42.9%)	28 (100.0%)	
Tunisia	20 (95.2%)	1 (4.8%)	21 (100.0%)	

Legend: * *p* < 0.05.

**Table 8:** Cumulative preventive deficit score.

	Romania (*n* = 189)	Tunisia (*n* = 160)	Total (*n* = 349)	*p*
Cumulative preventive deficit score, mean ± DS				<0.001 *
	2.77 ± 1.26	3.39 ± 1.08	3.05 ± 1.22	

Legend: * *p* < 0.05.

**Table 9:** Distribution of chronic treatment by country.

Therapeutic Class	Romania (*n* = 189)	Tunisia (*n* = 160)	Total (*n* = 349)	*p*
Any chronic treatment, *n* (%)	45 (23.8%)	62 (38.8%)	107 (30.7%)	0.004 *
Corticosteroid therapy	1 (0.5%)	12 (7.5%)	13 (3.7%)	0.002 *
Antiepileptics	1 (0.5%)	5 (3.1%)	6 (1.7%)	0.098 †
Anticancer drugs	2 (1.1%)	5 (3.1%)	7 (2.0%)	0.254 †
Proton Pump Inhibitors	35 (18.5%)	30 (18.8%)	65 (18.6%)	1.000
Antidepressants	6 (3.2%)	10 (6.2%)	16 (4.6%)	0.266
No chronic treatment	144 (76.2%)	98 (61.3%)	242 (69.3%)	-

Legend: * *p* < 0.05. Pearson’s Chi-square test was applied to 2 × 2 contingency tables (country × presence/absence of the respective class), except for those marked †, where the expected frequencies < 5 required the use of the Fisher Exact Test. For the overall pharmacological profile Chi^2^ = 21.13; df = 5; *p* < 0.001 (result to be interpreted with caution: 4/12 cells with expected frequencies < 5).

**Table 10:** Distribution of information sources between countries.

Source of Information	Romania (*n* = 141)	Tunisia (*n* = 97)	*p*
Physician	37 (26.2%)	54 (55.7%)	<0.001 *
Friends/family	36 (25.5%)	24 (24.7%)	
Social media	39 (27.7%)	14 (14.4%)	
Other sources	29 (20.6%)	5 (5.2%)	

Legend: * *p* < 0.05.

**Table 11:** Knowledge score by primary source of information (physician vs. other sources).

Sample	Physician, *n*, M ± DS	Other Sources, *n*, M ± DS	*p*
Global (*N* = 238)	91; 9.31 ± 2.58	147; 8.78 ± 2.21	*p* = 0.092
Romania (*n* = 141)	37; 9.51 ± 1.98	104; 9.02 ± 1.84	*p* = 0.171
Tunisia (*n* = 97)	54; 9.17 ± 2.93	43; 8.19 ± 2.86	*p* = 0.101

**Table 12:** Knowledge score based on receiving information from the physician.

Sample	Yes, *n*, M ± DS	No, *n*, M ± DS	*p*
Global (*N* = 349)	127; 8.96 ± 2.42	222; 8.09 ± 2.73	*p* = 0.003 *
Romania (*N* = 189)	74; 9.36 ± 1.86	115; 8.60 ± 2.36	*p* = 0.020 *
Tunisia (*N* = 160)	53; 8.40 ± 2.96	107; 7.55 ± 2.98	*p* = 0.093

Legend: * *p* < 0.05.

**Table 13:** Prevalence of risk factors and preventive behaviors in osteoporotic risk subgroup (patients 50 years of age).

Variable	Romania (*n* = 145)	Tunisia (*n* = 121)	*p*
Associated pathologies, *n* (%)	50 (34.5%)	45 (37.2%)	0.741
Chronic treatment, *n* (%)	39 (26.9%)	43 (35.5%)	0.166
Confirmed diagnosis of osteoporosis, *n* (%)	28 (19.3%)	21 (17.4%)	0.802
Fractures in history, *n* (%)	39 (26.9%)	22 (18.2%)	0.124
Family history of osteoporosis, *n* (%)	22 (15.2%)	42 (34.7%)	<0.001 *
Screening DXA, *n* (%)	41 (28.3%)	16 (13.2%)	0.005 *
Calcium supplements, *n* (%)	46 (31.7%)	27 (22.3%)	0.115
Vitamin D supplements, *n* (%)	60 (41.4%)	17 (14.0%)	<0.001 *
Active smoking, *n* (%)	27 (18.6%)	27 (22.3%)	0.553
Knowledge score, M ± DS	8.90 ± 2.20	7.83 ± 2.99	0.005 *
Preventive deficit score (0–5), M ± DS	2.65 ± 1.29	3.30 ± 1.12	<0.001 *

Legend: * *p* < 0.05. Tests: Chi-square for categorical variables; Welch *t*-test for continuous variables.

## Data Availability

The original contributions presented in this study are included in the article. Further inquiries can be directed to the corresponding authors.

## Supplementary Materials

The following supporting information can be downloaded at https://www.mdpi.com/article/10.3390/diseases14070235/s1, Supplementary Table S1. Harmonization of variables included in the pooled cross-national analysis.

## Abbreviations

The following abbreviations are used in this manuscript:

BMI	Body Mass Index
BMD	Bone Mineral Density
CPDS	Cumulative Preventive Deficit Score
DXA	Dual-Energy X-ray Absorptiometry
PPIs	Proton Pump Inhibitors
SSRIs	Selective Serotonin Reuptake Inhibitors
IBM	International Business Machines
SPSS	Statistical Package for the Social Sciences
CI	Confidence Interval
SD	Standard Deviation
OR	Odds Ratio
CPS	Cumulative Preventive Deficit Score
ES	Standard Error
BootCI	Bootstrap Confidence Interval
PROCESS	PROCESS Macro for Mediation, Moderation, and Conditional Process Analysis
R^2^	Coefficient of Determination
χ^2^	Chi-Square
df	Degrees of Freedom
*p*	Probability Value (*p*-value)
M	Mean
Beta	Standardized Regression Coefficient
Wald	Wald Test Statistic
t	*t*-test Statistic
*N*	Number of Participants/Sample Size
*n*	Number/Frequency
vs.	versus
IC	Confidence Interval
